# Clonal hematopoiesis of indeterminate potential is associated with increased risk of immune checkpoint inhibitor myocarditis in a prospective study of a cardio-oncology cohort

**DOI:** 10.1186/s40959-024-00289-z

**Published:** 2024-11-26

**Authors:** Rachel Jaber Chehayeb, Jaiveer Singh, Carlos Matute-Martinez, Nathan W. Chen, Ana Ferrigno Guajardo, Derrick Lin, Ritujith Jayakrishnan, Anthos Christofides, Etienne Leveille, Yunju Im, Giulia Biancon, Jennifer VanOudenhove, Eiman Ibrahim, Anastasias Ardasheva, Alokkumar Jha, John Hwa, Stephanie Halene, Jennifer M. Kwan

**Affiliations:** 1 Hospital of the University of Pennsylvania, Philadelphia, PA USA; 2 Yale School of Medicine, New Haven, CT USA; 3 Section of Cardiovascular Medicine, Yale University School of Medicine, New Haven, CT 06511 USA; 4 Yale College, New Haven, CT USA; 5 Yale New Haven Hospital, New Haven, CT USA; 6 Yale Cancer Center, New Haven, CT USA; 7 University of Nebraska Medical Center, Omaha, USA; 8 Section of Hematology, Department of Internal Medicine and Yale Cancer Center, Yale University School of Medicine, New Haven, CT USA; 9 Weill Cornell Medicine, New York, NY USA; 10 Department of Pathology, Yale University School of Medicine, New Haven, CT USA; 11 Yale School of Medicine, 300 George St #770B, New Haven, CT 06511 USA

**Keywords:** Clonal hematopoiesis, Clonal hematopoiesis of indeterminate potential, CHIP, Immunotherapy, ICI myocarditis, Neoplasms

## Abstract

**Background:**

Clonal hematopoiesis of indeterminate potential (CHIP) has been shown to increase all-cause mortality and risk of cardiomyopathy in patients with solid malignancies. CHIP has also been shown to increase T cell activation in heart failure patients. It is unclear whether CHIP can affect the risk of immune checkpoint inhibitor (ICI) myocarditis in patients with cancer treated with immunotherapy.

**Methods:**

We enrolled patients with solid tumors in a prospective study, determined CHIP status at time of enrollment through blood whole exome sequencing, and assessed incidence of ICI myocarditis from time of enrollment through December 1st, 2023. We performed a competing risk cox regression to evaluate the role of CHIP in ICI myocarditis, accounting for patient demographics, cardiac comorbidities, cardiotoxic cancer therapy, and dual ICI use in our covariates. We also generated cumulative incidence curves using subdistribution hazards to evaluate development of ICI myocarditis stratified by CHIP vs no CHIP. Chart review was performed to evaluate patient co-morbidities, lab values, imaging findings and outcomes.

**Results:**

Among the 88 patients receiving ICI therapy, average age was 67 ± 14 years, of which 50% harbored CHIP variants. Among all comorbidities, including diabetes, heart failure and obstructive coronary artery disease, only coronary artery calcifications were significantly increased in patients with CHIP. There were no statistically significant differences in cancer therapy or cardiovascular drugs between patients with and without CHIP. Among examined outcomes, patients with CHIP had a statistically higher rate of ICI myocarditis (overall: 57%, CHIP: 73% (32/44), no CHIP: 41% (18/44), *p* = 0.003) and death (CHIP: 60%, no CHIP 31%, *p* = 0.011). In a multivariate competing risk analysis, CHIP status doubled the risk of developing ICI myocarditis, similar to the risk of dual ICI use (CHIP status HR 2.74, 95% CI: 1.44–5.22, *p* = 0.002 vs dual ICI use HR 2.39, 95% CI: 1.11–5.14, *p* = 0.026).

**Conclusions:**

This study is the first to show that CHIP independently increases risk of ICI myocarditis, with implications for risk stratification of patients prior to ICI initiation and frequency of cardiac monitoring.

**Supplementary Information:**

The online version contains supplementary material available at 10.1186/s40959-024-00289-z.

## Background

Clonal hematopoiesis of indeterminate potential (CHIP) has been shown to increase all-cause mortality and risk of cardiomyopathy in patients with solid cancers [1, 2]. While immune checkpoint inhibitors (ICI) have greatly improved survival in several types of cancer, they are associated with ICI myocarditis, which has a fatality rate of up to 50%, especially in patients with higher grade toxicities [3]. Dual ICI use is the only consistent risk factor for ICI myocarditis [4]. Recently, single cell sequencing in heart failure patients showed that T cells harboring CHIP mutations are more proinflammatory and more activated compared to T cells without CHIP mutations [5]. In patients with DNMT3A driver mutations, single-cell sequencing revealed that monocytes, CD4 + T cells and natural killer (NK) cells carrying a DNMT3A mutation have altered gene expression profiles, with monocytes showing increased expression of inflammatory and phagocytosis genes, and T cells showing upregulation in activation and effector roles [6].

It remains unclear whether patients with CHIP receiving ICI therapy are at increased risk for ICI myocarditis. Hence, we sought to evaluate the risk of ICI myocarditis in patients with solid malignancies with and without CHIP treated with ICI at Yale New Haven Hospital.

## Methods

### Study design

In a prospective study approved by the Yale IRB, we enrolled patients undergoing treatment for solid tumors who were referred to cardio-oncology or seen by the cardiology service. Most patients either had underlying cardiovascular disease and were referred for closer monitoring and risk stratification or were admitted patients for whom cardio-oncology was consulted in the inpatient setting due to concern for cardiac toxicity. Reasons for referral included risk assessment for starting ICI, new cardiac symptoms including chest pain or dyspnea, elevated biomarkers such as troponin T, ECG changes, or changes in heart function. Some patients were prospectively monitored, enrolled prior to starting any immunotherapy, and did not develop ICI myocarditis.

### Data collection

We evaluated CHIP status by blood draw at time of enrollment and assessed the incidence of ICI myocarditis from the ICI start date to December1^st^, 2023. Of 236 total enrolled patients, 88 were previously on or receiving ICI treatment and were the focus of this study. ICI myocarditis was defined based on cMRI imaging as per the International Cardio-Oncology (IC-OS) definition for ICI myocarditis, based on the major criteria (diagnostic cardiac magnetic resonance imaging [cMRI] per the modified Lake Louise criteria) or the minor criteria combining clinical and cMRI findings recommended by the 2022 ESC guidelines [7, 8]. CMR criteria for myocarditis included increased T1 value of > 1000 ms or having positive late gadolinium enhancement and increased T2 signal > 60 ms on T2 mapping or having a myocardial to skeletal muscle ratio > 2 on T2-weighted short-tau inversion recovery [9]. CMR parameters of patients on ICI were pulled from radiology reports. Additionally, we classified patients into definite, probable, and possible myocarditis per the Bonaca criteria using a combination of CMR markers, biomarkers, EKG changes and clinical syndrome [10]. In addition, all patients received a thorough work up to rule out other or concurrent causes of myocarditis, including a thorough infectious work up and thyroid studies.

**Fig. 1:**
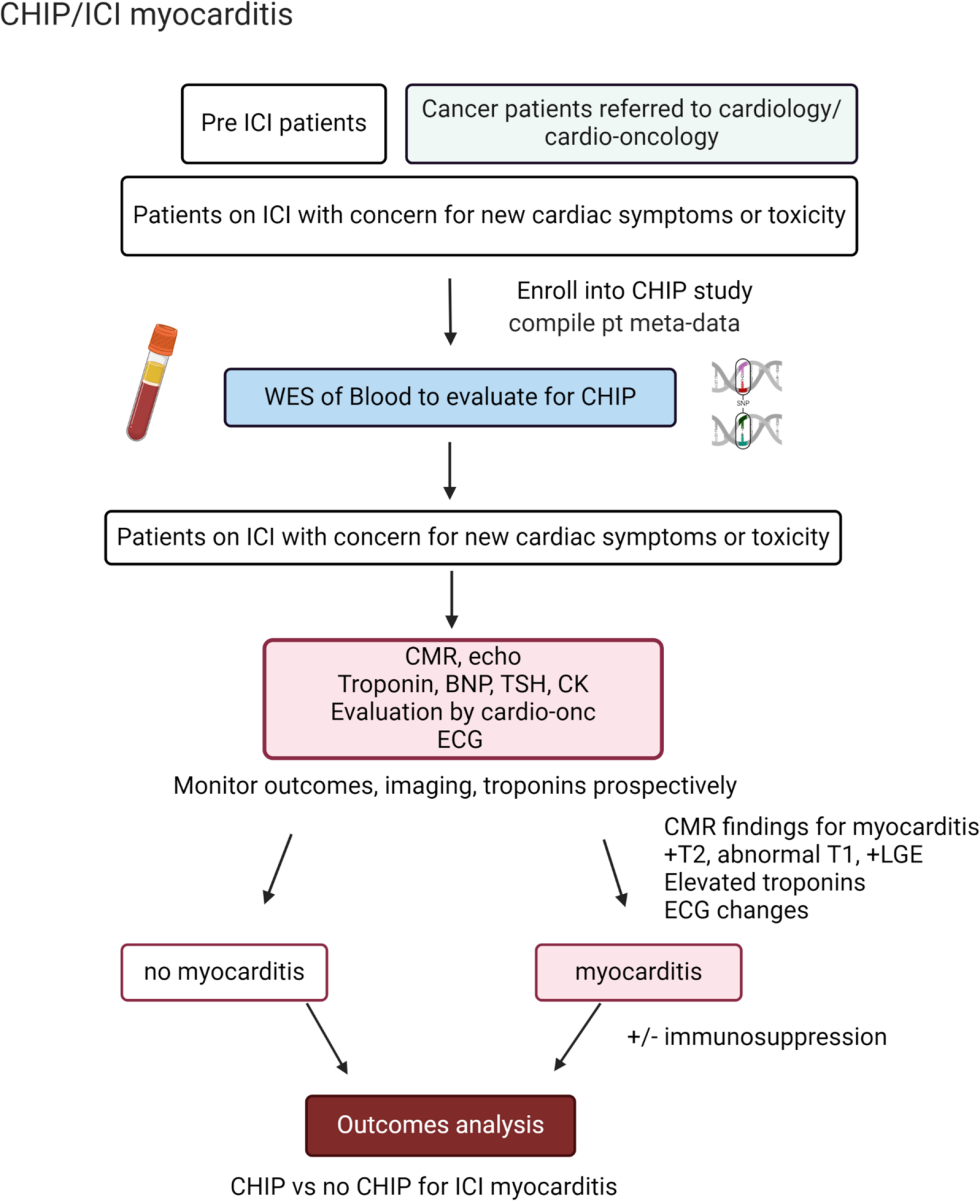
Flow Chart Summary of Our Prospective Observational Study of Enrolled Patients

Methodology to determine CHIP status and frequencies of CHIP mutations in our cohort have been previously described [2]. Continuous variables are presented as means with standard deviations, while categorical variables are shown as percentages. For comparisons based on CHIP status t-test or Fisher’s exact tests were used depending on the types of the variables. A p-value of 0.05 was used to declare statistical significance. We generated Kaplan Meier curves comparing time to ICI myocarditis development in our cohort stratified by CHIP status. Chart review was performed to evaluate patient co-morbidities, lab values, imaging findings and outcomes. Figure 1 provides a visual summary of our study design.

### Data analysis

A competing risk cox regression was performed to evaluate the role of CHIP in ICI myocarditis risk. Covariates of interest included patient demographics, co-morbidities including hypertension (HTN), hyperlipidemia (HLD), diabetes mellitus (DM), obstructive coronary artery disease (CAD), diabetes, prior chemotherapy exposure, cardiotoxic cancer therapy such as anthracycline, HER2 inhibitors, chest radiation (XRT), or use of dual ICI. Cumulative incidence curves using subdistribution hazards with gray’s test were also performed to evaluate development of ICI myocarditis stratified by CHIP vs no CHIP.

We also performed a secondary analysis examining time to myocarditis stratified by CHIP status among our subset of patients on dual ICI who developed myocarditis to assess for potential effect of CHIP in modifying myocarditis risk.

This study was approved by the Yale University IRB. The cox regression model and survival analysis were performed in R version 4.3.1 (2023–06–16), using ‘survival’ package (version survival_3.6–4).

## Results

Among the 88 patients receiving ICI therapy, average age was 67 ± 14 years (Table 1). The proportion of patients harboring CHIP variants was 50% (44/88), which is higher than in general cancer patients [11]. Supplementary Table 1 summarizes CHIP driver mutations and mutation frequency stratifying by variable allele fraction percentages, and Supplementary Table 2 summarizes the different immunotherapies received and their frequencies. The most frequent CHIP mutations include *DNMT3A* (14/44*), TET2* (8/44), *and PPM1D* (8/44)*.* The most frequent ICI prescribed include Pembrolizumab (50/88) and Nivolumab (27/88).

**Table 1:** Demographics and Clinical Characteristics of Patients receiving ICI Therapy Stratified by CHIP Status (*N* = 88)

**Variables**	**Total**, *N* = 88	**No CHIP**, *N* = 44	**CHIP**, *N* = 44	***P*-value**^1^
Age (Years)				
Mean (SD)	67 (14)	65 (13)	69 (15)	0.11
Gender				0.2
Female	42 (48%)	24 (55%)	18 (41%)	
Male	46 (52%)	20 (45%)	26 (59%)	
**Comorbidities**				
HLD	58 (66%)	32 (73%)	26 (59%)	0.2
HTN	57 (65%)	29 (66%)	28 (64%)	0.8
HFrEF	18 (20%)	9 (20%)	9 (20%)	> 0.9
HFpEF	14 (16%)	6 (14%)	8 (19%)	0.5
Diabetes	14 (16%)	8 (18%)	6 (14%)	0.6
CAD/CAC	56 (64%)	20 (45%)	36 (84%)	< 0.001
Metastasis	46 (52%)	23 (52%)	23 (52%)	> 0.9
Obstructive CAD	16 (18%)	10 (23%)	6 (14%)	0.3
**Cancer Therapy**				
Her2i	3 (9.7%)	2 (%)	1 (%)	0.6
XRT (chest)	37 (43%)	18 (42%)	19 (44%)	0.8
Anthracycline	33 (38%)	17(39%)	16 (38%)	> 0.9
Dual ICI	16 (18%)	7 (16%)	9 (21%)	0.5
**Cardiovascular Drugs**				
Beta Blocker	46 (56%)	19 (45%)	27 (68%)	0.042
ACE inhibitor/ ARB	28 (35%)	14 (33%)	14 (36%)	0.8
Calcium channel blockers	15 (19%)	8 (20%)	7 (18%)	0.9
Spironolactone	7 (8.2%)	4 (9.3%)	3 (7.1%)	> 0.9
Statin	46 (53%)	27 (61%)	19 (44%)	0.11
Sacubitril-valsartan	5 (6.2%)	3 (7.1%)	2 (5.1%)	> 0.9
**Outcomes**				
Cardiomyopathy (LVEF < 50% on TTE, < 57% on cMRI, or drop > 10%)^a^	42 (48%)	9 (20%)	33 (77%)	< 0.001
Myocarditis (ICI)	50 (57%)	18 (41%)	32 (73%)	0.003
Diastolic Dysfunction	19 (31%)	7 (27%)	12 (33%)	0.6
Death	36 (46%)	11 (31%)	25 (60%)	0.011
Arrhythmia	20 (27%)	6 (17%)	14 (36%)	0.06

*SD* standard deviation, *HLD* hyperlipidemia, *HTN* hypertension, *HFrEF* heart failure with reduced ejection fraction, *HFpEF* heart failure with preserved ejection fraction, *CAD* coronary artery disease, *CAC* coronary artery calcification, *Her2i* HER2 inhibitor, *ICI* immune check point inhibitor, *ARB* angiotensin receptor blocker, *LVEF* left ventricular ejection fraction

^a^CMR LVEF cut off of 57% for reduced EF is an internal metric used at YNHH to match back to an EF of 50% on TTE

^1^Wilcoxon rank sum test; Pearson’s Chi-squared test; Fisher’s exact test

CHIP patients were older than non-CHIP patients (CHIP: 69 ± 15 years, no CHIP 65 ± 13 years, p = 0.11). 48% of subjects were female (CHIP: 41%, no CHIP 55%, *p* = 0.2). All patients (88/88, 100%) had a baseline transthoracic echocardiogram (TTE) or cMRI performed within one month of blood draw, and 60/88 (68.1%) had a follow up echo or cMRI. There was a significantly higher prevalence of coronary artery calcifications (CAC) in CHIP patients (CHIP: 84%, no CHIP 45%, *p *< 0.001).

There were no statistically significant differences between the incidence of obstructive CAD as assessed by stress test, CT coronary or cardiac catheterization (CHIP: 14%, no CHIP 23%, *p* = 0.3), heart failure with reduced ejection fraction (HFrEF) (CHIP: 20%, no CHIP: 20%, *p* > 0.9) or heart failure with preserved ejection fraction (HFpEF) (CHIP: 19%, no CHIP: 14%, *p* = 0.6) between the two groups.

Additionally, the frequencies of diabetes (overall 16%, CHIP: 14%, no CHIP: 18%, p = 0.6), hypertension, (overall 65%, CHIP: 64%, no CHIP: 66%, *p* = 0.8) and hyperlipidemia (overall 66%, CHIP: 59%, no CHIP: 73%, *p* = 0.2) did not significantly differ between groups. Significantly more patients with CHIP were prescribed beta blockers (overall 56%, CHIP: 68%, no CHIP: 45%, *p* = 0.042). There were no statistically significant differences in frequencies of other cardiac medications prescribed (angiotensin-converting enzyme inhibitors [ACEi] / angiotensin II receptor blockers [ARBs], angiotensin receptor-neprilysin inhibitor, spironolactone, calcium channel blocker, ARNI, low dose aspirin, and statin).

Among both groups, the three most frequent cancers were lung 30% (CHIP: 27%, no CHIP: 32%,), genitourinary 26% (CHIP: 32%, no CHIP:20%), and melanoma 15% (CHIP:14%, no CHIP:16%,), respectively (Supplementary Table 3). Other cancer types represented included breast (13%), gastrointestinal (5%), head and neck (3.4%), non-melanoma skin cancer (3.4%) and sarcoma (2.3%). Assessing frequencies of cardiotoxic cancer therapies, we found that 9.7% of patients received HER2-targeting agents (CHIP: 5.9%, no CHIP: 14%, *p* = 0.6), 38% received anthracyclines (CHIP: 38%, no CHIP: 39%, *p* > 0.9), and 43% received chest radiation therapy (CHIP: 44%, no CHIP: 39%, *p* = 0.8). There were no significant differences in cancer types, frequency of metastatic disease (total: 52%, CHIP: 52%, no CHIP: 52%, *p* > 0.9), and frequencies of cardiotoxic cancer therapies received between patients with and without CHIP. Mortality among patients with CHIP was significantly higher during the duration of this study (CHIP: 60%, no CHIP: 31%, *p* = 0.011).

Of the 88 patients on ICI, 50 (57%) developed ICI myocarditis per the Lake Louise Criteria. The median time from ICI start date to ICI myocarditis development was 442 days (Q1-Q3: 95–881 days), while the median time from blood draw to ICI start data was 118 days (Q1-Q3: 37,399). Significantly more patients with CHIP than patients without CHIP developed ICI myocarditis, defined by clinical and radiographic evidence of myocarditis [12] (overall: 57%, CHIP: 73% no CHIP: 41%, *p* = 0.003). There was significantly more positive T2 (64% vs 15%, *p* < 0.001) or combined abnormal T1 and T2 (45% vs 15%, *p* = 0.011) and 2/2 Lake Louise Criteria (LLC) (54% vs 16%, *p* = 0.005) in those diagnosed with ICI myocarditis vs no myocarditis. Supplementary Table 4 summarizes the percentage of patients who met major and minor criteria for cMRI diagnosis of ICI myocarditis. Patients with myocarditis were also found to have a significantly higher median high sensitivity troponin T value (116 ng/L (IQR 30–530) in myocarditis vs 15 ng/L (IQR 3–69) in no myocarditis group, *p* < 0.001) with the upper limit of normal being 15 ng/L and a significantly higher rate of arrhythmias (46% in myocarditis group vs 13% no myocarditis group, *p* < 0.001). Among the 50 patients with ICI myocarditis, 19 (38%) had pre-existing cardiomyopathy (EF < 50% on transthoracic echocardiogram or > 57% on cardiac MRI) and 32 (64%) had worsening cardiomyopathy.

Per Bonaca criteria, among the 50 patients diagnosed with ICI myocarditis, 74% were definite, 18% were probable, and 8% were possible myocarditis (Supplementary Table 5). In summary, 80% of patients presented with dyspnea on exertion, 88% had elevated high sensitivity troponin T levels, 24% had EKG changes and 78% had worsening cardiomyopathy. At least 1 / 2 LLC criteria for myocarditis were presented in 76% of patients, as summarized in Supplementary Table 4.

Of the 42 patients that were treated with steroids, 36 (~ 86%) responded to steroids or to escalation of immunosuppression, as defined by improvement in at least one of the Bonaca criteria, with 81% having down-trending high sensitivity troponin T, 5% having resolution of EKG changes, and 21% exhibiting improvement in imaging findings on follow up cMRI, supporting the cMRI-based diagnosis of ICI myocarditis. Supplementary Table 6 summarizes the reasoning for deferring steroids in the 8 patients with untreated myocarditis.

Overall, 29 patients (58%) had ischemic evaluation, with 20% (10 patients) receiving CT coronary, 28% (14) undergoing LHC, and 10% (5) undergoing some stress testing. Of the 29, six patients had evidence of obstructive CAD defined by left main stenosis > 50% or stenosis of any other vessel > 70% [13]. Of the six, five responded to steroids. Among the 21 patients with no ischemic evaluation, 13 were classified as having definite myocarditis by Bonaca criteria, six as having probable myocarditis, and two as having possible myocarditis. Of the 21, 7 did not receive steroids (Supplementary Table 6). Of the remaining 14 patients, 13 improved with steroids (down trending troponin T and clinical syndrome improvement), and one patient had minimal improvement and was transitioned to comfort measures only.

For the 4 patients with no myocarditis that had 2/2 LLC criteria for myocarditis seen in Supplementary Table 4, they either had an ischemic event or known cMRI abnormalities prior to ICI treatment initiation and were not treated with immunosuppression.

**Fig. 2:**
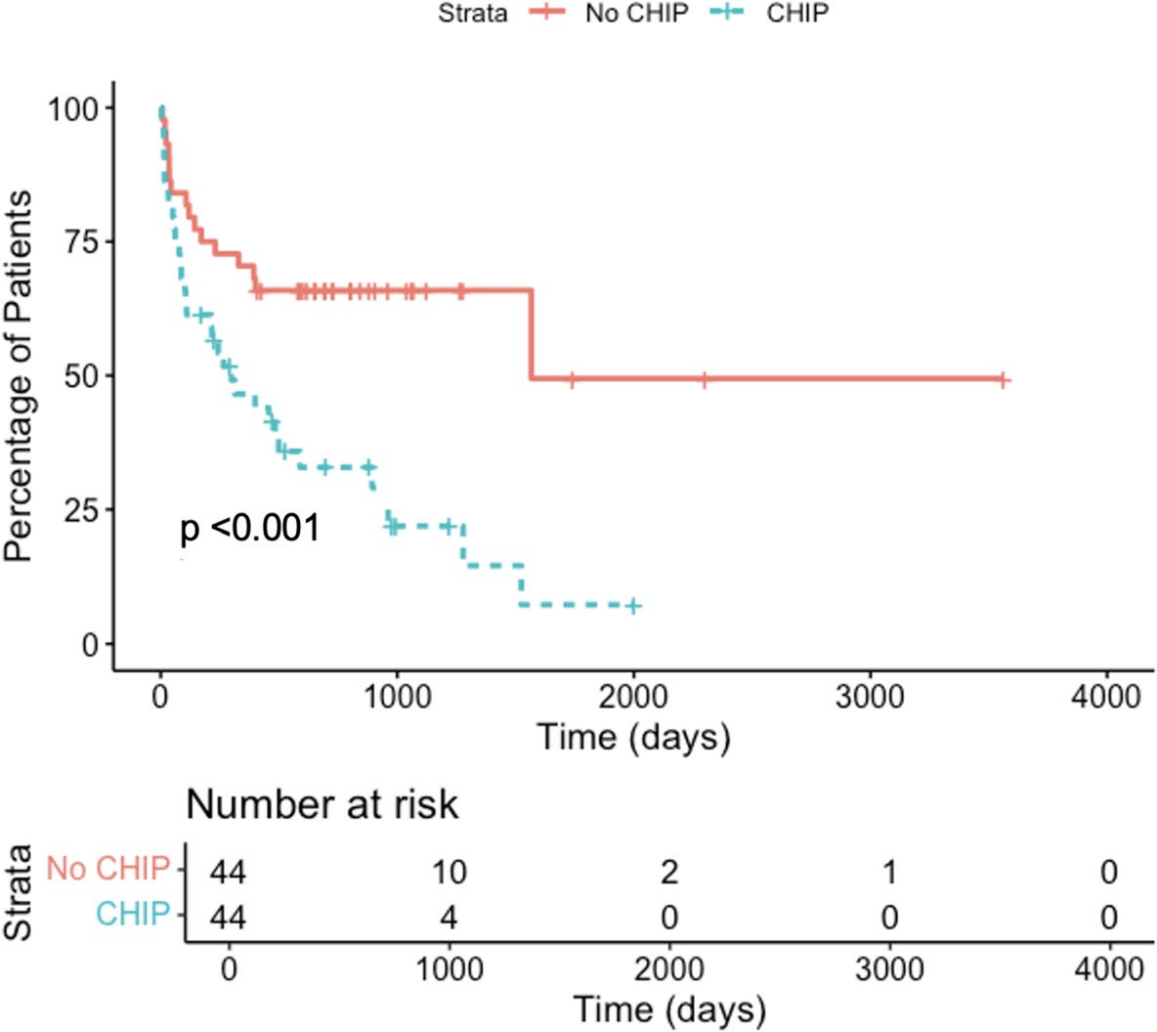
KM curves comparing CHIP vs no CHIP for ICI myocarditis development

**Fig. 3:**
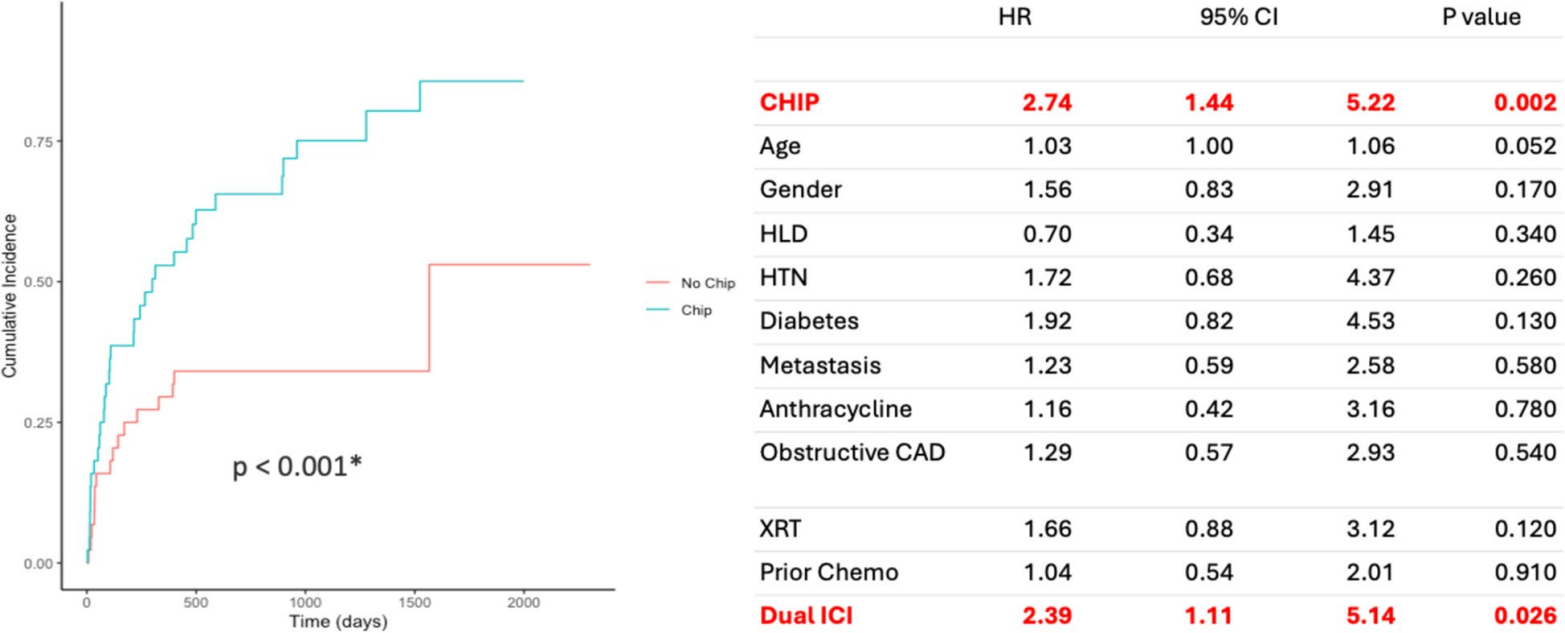
CHIP is associated with the increased risk of ICI myocarditis. Cumulative incidence curves showing the development of ICI myocarditis by CHIP status (left) Competing risk multivariable cox regression model for ICI myocarditis (right).  *by Gray’s test.CHIP= clonal hematopoiesis of indeterminate potential, HR= hazard ratio, CI= confidence interval, HLD= hyperlipidemia, HTN= hypertension, CAD= coronary artery disease, ICI=immune check point inhibitor, XRT= radiation therapy

As shown in Fig. 2, patients with CHIP had a shorter time to develop ICI myocarditis compared to patients without CHIP (median time of 255 (Q1 70, Q3 644) days IQR 543 days vs 635 (Q1 201, Q3 934) days IQR 705, respectively; *p* = 0.00049). Cumulative incidence curves using subdistribution hazards revealed a significant difference in ICI myocarditis incidence between patients with and without CHIP by Gray’s test Fig. 3 (left). In a multivariate competing risk analysis, CHIP status doubled the risk of developing ICI myocarditis, similar to the risk of dual ICI use (CHIP status HR 2.74, 95% CI: 1.44–5.22, *p* = 0.002 vs dual ICI use HR 2.39, 95% CI: 1.11–5.14, *p* = 0.026), Fig. 3 (right).

We performed a secondary analysis examining time to myocarditis stratified by CHIP status amongst a subset of patients on dual ICI who developed myocarditis (N = 16). Of the 16 patients on dual ICI, 9/16 (56.25%) had CHIP. In this subpopulation, there were no significant differences in cancer types or cardiotoxic therapies between patients with CHIP and no CHIP (Supplementary Table 7). Patients with CHIP had a significantly higher frequency of coronary artery calcifications than patients without CHIP (CHIP 78%, no CHIP 14%, *p* = 0.041). Patients with CHIP had a shorter time to develop ICI myocarditis, although this finding was not statistically significant (median time of 216 days IQR 16–299 days vs 600 days IQR 43–1265, respectively; *p* = 0.093), as shown in Supplementary Fig. 1.

## Discussion

To our knowledge, our work is the first to report that CHIP is associated with an increased risk of ICI myocarditis among patients with solid malignancies being treated with immunotherapy. While rare, ICI myocarditis is a disease with a high mortality rate (up to 50%) especially if presenting late or with a higher grade toxicity, and so far, only dual ICI use has been identified as a consistent risk factor [3]. Our findings of an additional risk factor for ICI myocarditis, CHIP, could hence be critical in risk stratifying patients prior to initiation of single or dual ICI therapy.

Over the past decade, there has been on-going research to optimize the diagnostic criteria for ICI myocarditis. Our study uses both the Lake-Louise Criteria (LLC) as well as the Bonaca criteria to establish the diagnosis of ICI myocarditis. Lake-Louise criteria have been shown to have limited sensitivity for ICI-myocarditis. In a 2020 review of ICI myocarditis, the authors note the phenomenon of LGE negative myocarditis, citing 2 different studies of patients with a diagnosis of ICI myocarditis and no evidence of LGE on imaging (8/31 patients in one study and 10/13 in another study) [4, 14, 15]. The authors attributed this to early imaging or incorrect clinical diagnosis. In a 2019 study by Zhang et al., among 56 pathology-proven cases of ICI-associated myocarditis who underwent cMRI, around half (48%) had negative LGE, and negative T2 STIR [16]. Given the limited sensitivity of the LLC, we also incorporated the Bonaca criteria and showed improvement in at least one set of criteria (cMRI findings, EKG changes, elevated troponin T) in the vast majority of patients treated with steroids (86%), supporting the empiric diagnosis of ICI myocarditis [10]. Molecularly targeted nuclear imaging, such as CD-8 PET, is a promising future approach that would be more sensitive and specific than cMRI-based LLC [17].

In the absence of endomyocardial biopsies, we are unable to determine whether all myocarditis diagnosed by Lake-Louise and Bonaca criteria are true T-cell mediated autoimmune lymphocytic myocarditis. While endomyocardial biopsy is considered the gold standard for diagnosing ICI myocarditis, the Dallas criteria for histopathological diagnosis have been associated with poor sensitivity given the patchy nature of ICI myocarditis and intra-observer variability carries higher risk than noninvasive techniques [8, 18]. A recent retrospective study of patients with ICI myocarditis who underwent endomyocardial biopsy reported that only 2/17 biopsies satisfied the Dallas criteria for ICI myocarditis [18]. Hence, in our institutional practice, endomyocardial biopsies are reserved for cases of diagnostic ambiguity, after assessing empiric response to steroids and in the setting of high clinical suspicion for ICI myocarditis.

Further work is needed to determine the pathophysiology of how CHIP increases the risk of developing ICI myocarditis. As mentioned in the introduction, single cell sequencing has shown that T cells harboring CHIP mutations are more inflammatory, with altered gene expression profiles and upregulation in activation and effector roles, and with increased expression of phagocytosis genes in monocytes [5, 6]. These findings provide potential building blocks for further research into the pathophysiology of CHIP and ICI myocarditis risk specifically. Further additional studies including spatial-omics techniques and correlation with biopsies and autopsies to determine what other cell types are involved or if CHIP’s role is mediated primarily through T cells.

Of note, recent studies have also shown that, in patients with CHIP and heart failure, CHIP mutated monocytes with DNMT3A driver mutations interact with cardiac fibroblasts and potentially increase cardiac fibrosis through increased release of heparin-binding epidermal growth factor-like growth factor that mediates cardiac fibroblast activation [19]. Hence, it is possible that cardiac fibrosis is a potential confounder in patients having positive DGE or increased T1 mapping, which is found in 62% of our patients meeting criteria for myocarditis (Supplementary Table 4) [20]

However, fibrosis should not influence T2 positivity, which is present in 64% of patients with myocarditis in our sample [18]. Our group has previously shown that there is increased DGE in cardio-oncology patients with CHIP compared to no CHIP [21].

Another potential confounder is HER2 inhibitors, which can cause an increase in T2 in patients in the absence of significant cardiomyopathy [22]. However, only 3 patients in our study were on HER2 inhibitors, and none of those 3 patients developed ICI myocarditis.

### Limitations

Our study has several limitations. Most of our patients were referred to cardiology, hence the baseline incidence of cardiac comorbidities in our patient population, for example, coronary artery disease, is higher than in the overall population, or in a more general oncology cohort. In addition, this is a single center cohort, and larger multicenter cohorts would allow for further validation of the effect of CHIP on ICI myocarditis. Moreover, given the smaller size of our cohort, we did not have enough statistical power to assess the effect of CHIP on ICI myocarditis development in patients specifically receiving dual ICI. Another limitation includes the apparent deviation in mortality rates of ICI myocarditis, with most patients dying within the first 30 days in the reported literature, as opposed to the prolonged survival seen in Kaplan Meier curves in Fig. 2 with survival curves separating further along in the 100–200 day range [3]. One explanation for these differences is the variations in grades of toxicity with most of our patients having grade 2–3 toxicity instead of grade 4 fulminant toxicity which is associated with the high immediate mortality. Another contributing factor could be close surveillance of troponin T in patients referred to cardio-oncology allowing for earlier detection and treatment.

## Conclusions

In summary, this study is the first to show that CHIP independently increases the risk of ICI myocarditis. Our study used 1) both the LLC and Bonaca criteria, combining cMRI findings with cardiac biomarkers, EKG changes, and clinical symptoms, 2) thorough work up to rule out other causes of cardiac inflammation and worsening cardiac function, including ischemic evaluation and thyroid testing, and 3) empiric response to steroids to diagnose myocarditis with high accuracy. ICI myocarditis was likely higher in this cohort compared to a general population of patients on ICI due to the patients being referred to cardiology for cardiac reasons or concern for cardiotoxicity. Identifying CHIP as a risk factor for ICI myocarditis has potential implications for risk stratification of patients prior to ICI initiation, clinical decision making, and frequency of cardiac monitoring.

## Supplementary Information

Supplementary Material 1.

## Data Availability

The data generated in this study are not publicly available due to IRB stipulations but are available upon reasonable request from the corresponding author.

## Abbreviations

ICI: Immune check point inhibitor
CAD: Coronary artery disease
HFrEF: Heart failure with reduced ejection fraction
HFpEF: Heart failure with preserved ejection fraction
ARB: Angiotensin receptor blocker
HER2: Human epidermal growth factor receptor 2
HTN: Hypertension
HLD: Hyperlipidemia
CCB: Calcium channel blocker
CM: Cardiomyopathy
CHIP: Clonal hematopoiesis of indeterminate potential
CAC: Coronary artery calcifications
LLC: Lake Louise Criteria
ISR: Immunosuppression resistant
