# Supplementary material for: Clonal hematopoiesis of indeterminate potential is associated with increased risk of immune checkpoint inhibitor myocarditis in a prospective study of a cardio-oncology cohort
Source: Cardiooncology. 2024 Nov 26;10:84. doi: 10.1186/s40959-024-00289-z (PMC11590368; doi:10.1186/s40959-024-00289-z)
Supplement: Supplementary file 1 — Supplementary Material 1. [file 40959_2024_289_MOESM1_ESM.docx]

**Supplementary Material Outlines**

1. **Supplementary Table 1:** Distribution of CHIP Driver Mutations and Stratification by Variable Allele Fraction
2. **Supplementary Table 2:** Immunotherapies Patients Received and Associated Frequencies
3. **Supplementary Table 3:** Cancer types Stratified by CHIP Status
4. **Supplementary Table 4:** CMR characteristics for those diagnosed and treated for ICI myocarditis vs those without ICI myocarditis.
5. **Supplementary Table 5:** Bonaca Criteria and Prevalence of Individual Criteria Among Patients with ICI Myocarditis
6. **Supplementary Figure 1:** KM curves comparing CHIP vs no CHIP for ICI myocarditis development among the dual ICI Subset
7. **Supplementary Table 6**: Clinical and Radiographic Data and Reasons for Deferring Immunosuppression in Patients with Untreated Myocarditis
8. **Supplementary Table 7:** Demographics and Clinical Characteristics and Outcomes of Patients receiving Dual ICI Therapy Stratified by CHIP Status

**Supplementary Table 1: Distribution of CHIP Driver Mutations and Stratification by Variable Allele Fraction (VAF)**

| **Gene** | **n** | VAF 2-5% | VAF 5-10% | VAF 10-20% | VAF >20% |
| --- | --- | --- | --- | --- | --- |
| DNMT3A | 14 | 8 | 3 | 2 | 1 |
| TET2 | 8 | 3 | 3 |  | 2 |
| PPM1D | 8 | 6 | 1 | 1 |  |
| ASXL1 | 5 | 2 |  |  | 3 |
| EZH2 | 4 | 4 |  |  |  |
| CBL | 3 | 3 |  |  |  |
| JAK2 | 2 | 1 |  |  | 1 |
| TP53 | 1 | 1 |  |  |  |
| IDH2 | 1 |  |  |  | 1 |
| U2AF1 | 1 | 1 |  |  |  |
| SF3B1 | 1 | 1 |  |  |  |
| NOTCH1 | 1 |  |  |  | 1 |
| NRAS | 1 | 1 |  |  |  |
| FBXW7 | 1 |  |  |  | 1 |
| KDM6A | 1 |  |  |  | 1 |
| KMT2B | 1 |  |  |  | 1 |
| BCL11B | 1 | 1 |  |  |  |

**Supplementary Table 2:** Immunotherapies Patients Received and Associated Frequencies.

ICI= immune check point inhibitor

| **ICI** | **Count** |
| --- | --- |
| Pembrolizumab | 50 |
| Atezolizumab | 8 |
| Nivolumab | 27 |
| Ipilimumab | 18 |
| Durvalumab | 9 |
| cemiplimab | 2 |
| tremelimumab | 1 |
| **avelumab** | 1 |

**Supplementary Table 3:** Cancer types Stratified by CHIP Status

| **Characteristic** | **Overall**, N = 88*^1^* | **No CHIP** N = 44*^1^* | **CHIP**  N = 44*^1^* | **p-value***^2^* |
| --- | --- | --- | --- | --- |
| Cancer Type |  |  |  | 0.7 |
| Breast | 11 (13%) | 7 (16%) | 4 (9.1%) |  |
| GI | 7 (8.0%) | 4 (9.1%) | 3 (6.8%) |  |
| GU | 23 (26%) | 9 (20%) | 14 (32%) |  |
| H&N | 3 (3.4%) | 1 (2.3%) | 2 (4.5%) |  |
| Lung | 26 (30%) | 14 (32%) | 12 (27%) |  |
| Melanoma | 13 (15%) | 7 (16%) | 6 (14%) |  |
| Sarcoma | 2 (2.3%) | 0 (0%) | 2 (4.5%) |  |
| Skin | 3 (3.4%) | 2 (4.5%) | 1 (2.3%) |  |
| *^1^* n (%) |  |  |  |  |
| *^2^* Fisher’s exact test |  |  |  |  |

**Supplementary Table 4:** CMR characteristics for those diagnosed and treated for ICI myocarditis vs those without ICI myocarditis.

DGE= delayed gadolinium enhancement, LLC= lake Louise criteria, LVEF= left ventricular ejection fraction, WMA= wall motion abnormalities.

^1^n (%), ^2^Pearson’s Chi-squared test; Fisher’s exact test

*EF cut off of 57% for reduced EF is an internal metric used at YNHH to match back to an EF of 50% on TTE.

| **Characteristic** | **N** | **No ICI Myocarditis**,  N = 26*^1^* | **ICI Myocarditis**,  N = 42*^1^* | **p-value***^2^* |
| --- | --- | --- | --- | --- |
| Positive_T1_or_DGE | 68 |  |  | 0.7 |
|  |  | 15 (58%) | 26 (62%) |  |
| Positive_T2 | 68 |  |  | <0.001 |
|  |  | 4 (15%) | 27 (64%) |  |
| Positive_Both | 68 |  |  | 0.011 |
|  |  | 4 (15%) | 19 (45%) |  |
| DGE ischemic, nonischemic, both | 38 |  |  | >0.9 |
| ischemic |  | 1 (7.7%) | 2 (8.0%) |  |
| nonischemic |  | 11 (85%) | 22 (88%) |  |
| nonischemic and ischemic |  | 1 (7.7%) | 1 (4.0%) |  |
| LLC 0/2, 1/2, 2/2 | 66 |  |  | 0.005 |
| 0/2 |  | 6 (24%) | 3 (7.3%) |  |
| 1/2 |  | 15 (60%) | 16 (39%) |  |
| 2/2 |  | 4 (16%) | 22 (54%) |  |
| LVEF<57% or WMA?* | 66 |  |  | 0.5 |
|  |  | 9 (36%) | 18 (44%) |  |

**Supplementary Table 5:** Bonaca Criteria and Prevalence of Individual Criteria Among Patients with ICI Myocarditis

***including 2 patients with COPD which could be a confounder**

**** see cMRI criteria from Supplementary Table 4**

|  | **N of patients** | **%** |
| --- | --- | --- |
| **Clinical Syndrome: Dyspnea on exertion / fatigue*** | 40 | 80 |
| **Clinical Syndrome: Chest pain / tightness** | 11 | 22 |
| **Clinical Syndrome: Lower extremity edema** | 11 | 22 |
| **Troponin Elevation** | 44 | 88 |
| **EKG changes** | 12 | 24 |
| **Worsening cardiomyopathy** | 39 | 78 |
| **Cardiac MRI c/w myocarditis**** | 38 | 76 |
| ***Definite*** | 37 | 74 |
| ***Probable*** | 9 | 18 |
| ***Possible*** | 4 | 8 |

**Supplementary Figure 1:** Kaplan Meier curves comparing CHIP vs no CHIP for ICI myocarditis development among the dual ICI Subset

**
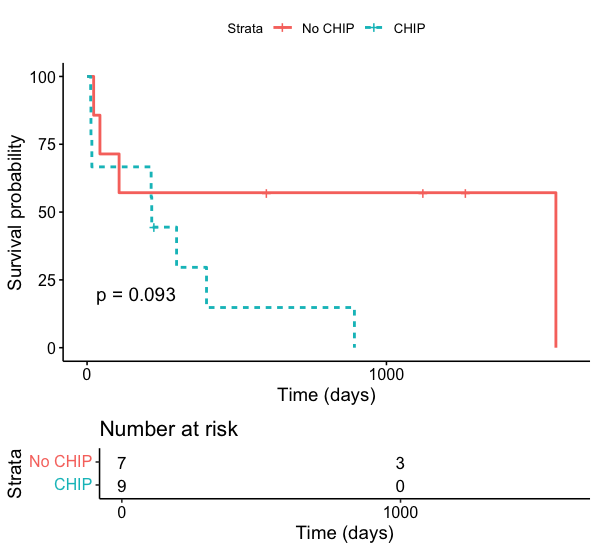
**

**Supplementary Table 6**: Clinical and Radiographic Data and Reasons for Deferring Immunosuppression in Patients with Untreated Myocarditis

| **CHIP patient** | **CMR findings/troponin** | **Reason for deferring immunosuppression** |
| --- | --- | --- |
| 1 | Had abnormal T2, nonischemic DGE reduced LVEF 52%, mildly elevated Trop T 0.05 | Advanced disease, deferred immunosuppression |
| 2 | Had abnormal T2, LVEF of 29%, Trop T 0.21 | Concern for septic shock |
| 3 | CMR not diagnostic due to PPM, LVEF drop to 31%, HST positive | Interdisciplinary discussion to hold off on steroids |
| 4 | Reduced LVEF, abnormal T1, no obvious increased T2, increased troponin | Interdisciplinary discussion to hold off on steroids |
| 5 | CMR with nonischemic DGE, severely depressed LVEF, elevated troponins | Interdisciplinary discussion to hold off on steroids |
| 6 | CMR nondiagnostic, LVEF reduced, elevated HS troponin 30s | Interdisciplinary discussion to hold off on steroids |
| 7 | CMR reduced quality, RV function reduced, Elevated troponins | Interdisciplinary discussion to hold off on steroids, concern for pneumonia |
| 8 | TTE reduced LVEF and RVEF, cannot do CMR due to shrapnel, a fib, elevated troponins | Interdisciplinary discussion to hold off on steroids |

**Supplementary Table 7:** Demographics and Clinical Characteristics and Outcomes of Patients receiving Dual ICI Therapy Stratified by CHIP Status

*^1^* Wilcoxon rank sum test; Fisher’s exact test

SD= standard deviation, HLD= hyperlipidemia, HTN= hypertension, HFrEF= heart failure with reduced ejection fraction, HFpEF= heart failure with preserved ejection fraction, CAD= coronary artery disease, CAC= coronary artery calcification, Her2i= HER2 inhibitor, ICI=immune check point inhibitor, ARB= angiotensin receptor blocker LVEF= left ventricular ejection fraction

| **Characteristic** | **Total**, N = 16 | **No CHIP**, N = 7 | **CHIP**, N = 9 | **p-value^1^** |
| --- | --- | --- | --- | --- |
| Age (Years) |  |  |  |  |
| Median (IQR) | 75 (55, 78) | 73 (49, 80) | 76 (61, 78) | 0.8 |
| Mean (SD) | 67 (17) | 65 (18) | 68 (18) | 0.8 |
| Gender |  |  |  | 0.3 |
| Female | 6 (38%) | 4 (57%) | 2 (22%) |  |
| Male | 10 (63%) | 3 (43%) | 7 (78%) |  |
| **Comorbidities** |  |  |  |  |
| HLD | 9 (56%) | 4 (57%) | 5 (56%) | >0.9 |
| HTN | 9 (56%) | 3 (43%) | 6 (67%) | 0.6 |
| HFrEF | 2 (13%) | 0 (0%) | 2 (22%) | 0.5 |
| HFpEF | 4 (25%) | 2 (29%) | 2 (22%) | >0.9 |
| Diabetes | 1 (6.3%) | 1 (14%) | 0 (0%) | 0.4 |
| CAD/CAC | 8 (50%) | 1 (14%) | 7 (78%) | 0.041 |
| Metastasis | 7 (44%) | 2 (29%) | 5 (56%) | 0.4 |
| Obstructive CAD | 0 (0%) | 0 (0%) | 0 (0%) | >0.9 |
| **Cancer Therapy** |  |  |  |  |
| Her2i | 0 (0%) | 0 (NA%) | 0 (0%) | >0.9 |
| XRT (chest) | 7 (44%) | 2 (29%) | 5 (56%) | 0.4 |
| Anthracycline | 1 (25%) | 0 (NA%) | 1 (25%) | >0.9 |
| **Cardiovascular Drugs** |  |  |  |  |
| Beta Blocker | 9 (60%) | 1 (14%) | 8 (100%) | 0.001 |
| ACE inhibitor. ARB | 7 (50%) | 3 (43%) | 4 (57%) | >0.9 |
| Calcium channel blockers | 2 (14%) | 1 (14%) | 1 (14%) | >0.9 |
| Spironolactone | 0 (0%) | 0 (0%) | 0 (0%) | >0.9 |
| Statin | 10 (63%) | 4 (57%) | 6 (67%) | >0.9 |
| Sacubitril-valsartan | 0 (0%) | 0 (0%) | 0 (0%) | >0.9 |
| **Outcomes** |  |  |  |  |
| Cardiomyopathy (LVEF <50% on TTE, 57% on cMRI, or drop >10%) | 10 (63%) | 3 (43%) | 7 (78%) | 0.3 |
| Myocarditis (ICI) | 12 (75%) | 4 (57%) | 8 (89%) | 0.3 |
| Diastolic Dysfunction | 4 (33%) | 0 (0%) | 4 (44%) | 0.5 |
| Death | 8 (50%) | 2 (29%) | 6 (67%) | 0.3 |
| Arrhythmia | 2 (13%) | 0 (0%) | 2 (25%) | 0.5 |
